# Heterosexual men who patronise entertainment establishments versus brothels in an Asian urban setting – which group practises riskier sexual behaviours?

**DOI:** 10.1186/s12889-015-2132-4

**Published:** 2015-08-14

**Authors:** Raymond Boon Tar Lim, Mee Lian Wong, Poh Huat Tan, Mandy Govender

**Affiliations:** Saw Swee Hock School of Public Health, National University of Singapore, Tahir Foundation Building, 12 Science Drive 2, 117549 Singapore, Singapore; Duke-National University of Singapore Graduate Medical School, 8 College Road, 169857 Singapore, Singapore; Health Promotion Board, 3 Second Hospital Avenue, 168937 Singapore, Singapore

## Abstract

**Background:**

Sex work has shifted from brothels to entertainment establishments (EEs) in Asia. Men who patronise EEs could act as a bridging population for human immunodeficiency virus (HIV) and sexually transmitted infection (STI) transmission through unprotected sex with the female EE workers to their spouses and regular partners. The aim of this study was to compare the prevalence and factors associated with risky sexual behaviours among the heterosexual men who patronised the EEs and brothels in Singapore.

**Methods:**

This was a cross-sectional survey involving 569 heterosexual men (297 recruited from brothels and 272 from EEs). A 2-stage sampling involving proportional stratified random sampling of the brothels and EEs, followed by time location sampling of the men, was conducted. For multivariable analysis, we used a mixed effects logistic model with backward elimination to account for clustering by venue and to obtain the adjusted odds ratio (aOR) for the association of various factors with consistent condom use in vaginal and oral sex respectively.

**Results:**

Men who patronised EEs were younger, more likely to be single, more highly-educated and comprised more professionals compared to the brothel group. On multivariable analysis, consistent condom use for vaginal sex decreased at the EE setting (aOR 0.64; 95 % CI: 0.42 –0.97) and with alcohol use before sex (aOR 0.67; 95 % CI: 0.46 – 0.98) and increased with perceived high risk of getting HIV/STIs from partner (aOR 2.08; 95 % CI: 1.30 – 3.32) and partner’s request for condom use (aOR 5.48; 95 % CI: 1.20 – 25.11). For consistent condom use with oral sex, this decreased at the EE setting (aOR 0.64; 95 % CI: 0.39 – 0.98) and with alcohol use before sex (aOR 0.50; 95 % CI: 0.31 – 0.81) and increased with partner’s request for condom use (aOR 5.19; 95 % CI: 1.38 – 19.57).

**Conclusions:**

Men who patronised EEs practised risker sexual behaviours compared to the brothel group. Priority should be given for intervention programmes to target men who patronise EEs, which could involve the female EE workers, the EE owners as well as the managers for effective HIV/STI prevention.

## Background

Worldwide, South and Southeast Asia is the second highest region affected by human immunodeficiency virus (HIV) after Sub-Saharan Africa [1]. In 2012, 3.7 million adults lived with HIV and 250,000 new cases of infection aged at least 15 years were reported from South and Southeast Asia [1]. In addition, the highest global burden of curable sexually transmitted infections (STIs) is also in this region [2]. Heterosexual intercourse is the main mode of HIV/STI transmission in South and Southeast Asia [2]. In recent years, casual sex has become more common in Asia, contributing to another important mode of transmission apart from male patronage of female sex workers [3–5]. For example, female casual partners have replaced female sex workers as the largest group of STI primary contacts among heterosexual males in Singapore [6, 7].

The social landscape of sex work has rapidly changed in Asia. There is an increasing shift of sex work from traditional brothel settings to entertainment establishments (EEs) [8]. These are places where people socialise with one another and engage in entertainment activities such as singing, dancing and drinking [8]. Typical EE settings include karaoke lounges, bars, pubs, nightclubs and discotheques where both commercial and casual sex occur [8]. Globalisation and income disparities in Asia have led to an influx of women to work in EEs. This coupled with a growing demand from heterosexual men have resulted in the recent surge of EEs in various parts of Asia, particularly in China, Vietnam, Thailand, Philippines and Singapore [8]. It was reported that up to 80 % of the female EE workers in China were involved in commercial sex, and more than 50 % of them had unprotected sex with their clients [9].

Previous studies on EEs have focused on the sexual behaviour of female EE workers, rather than men who patronise the EEs. This is an important and yet often neglected group because these men could also potentially act as a bridging population for HIV/STI transmission through unprotected sex with the female EE workers to their spouses and regular partners, resulting in extensive transmission to the community. In Tijuana, Mexico, men from EEs were significantly more likely to report unprotected paid vaginal or anal sex (odds ratio (OR) 2.27; 95 % confidence interval (CI): 1.40 – 3.67) [10] compared to men who met female sex workers on the streets. In addition, men from EEs were also significantly more likely to report having female casual partners (OR 1.81; 95 % CI: 1.08 – 3.01) [10] versus men who met female sex workers on the streets. Similarly in a large eastern city in China, men who had ever visited the EEs were more than 4 times as likely to report unprotected vaginal sex with a non-spouse female partner in the EEs (OR 4.25; 95 % CI: 3.15 –5.73) [9] than those who had never visited the EEs.

The few available studies on men who patronise EEs have focused on paid sex with the female EE workers and examined vaginal or anal intercourse only [9–11]. Casual sex is usually not the focus in these studies, although there is reason to believe that substantial casual sex also takes place in the EEs [12]. In addition, these studies did not look into oral intercourse. Unprotected oral intercourse can lead to viral STIs such as genital warts and herpes simplex type 1 infection [13], bacterial STIs such as pharyngeal gonorrhoea, syphilis, chancroid and Chlamydia trachomatis infection [14] and even HIV infection [15, 16]. To our knowledge, there is no published study in an Asian urban setting that compares the risky sexual behaviours among the heterosexual men who engage in different types of sexual intercourse in the EE and brothel-based settings, including oral sex with paid and casual partners. Compared to brothels which are often regulated and where there is access to condoms and HIV/STI screening and treatment services, sex work in the EEs is mostly hidden and illegal, often occurring under the influence of alcohol and with lack of access to these services [17–21]. It is critical to address these gaps in knowledge so that policy-makers, public healthcare professionals and clinicians can use the information to tailor their intervention programmes accordingly for better HIV/STI control.

Singapore, an urbanised city state in Southeast Asia, has in recent years also experienced an influx of women from other countries to work in the EEs. Other than the traditional brothels in the designated red-light districts, there is an increasing trend of heterosexual men who have paid or casual sex with female EE workers [22, 23]. Most of these female EE workers are on social visit passes, and are not employed by the EE owners nor do they have any direct association with the EEs. Anyone above the age of 18 can enter the EEs in Singapore. The EE owners allow entry of the female EE workers because they attract men to patronise the EEs and spend. The sources of income for these female EE workers include tips when they chat, sing, dance and drink with the men, commission from the alcoholic drinks and the provision of sexual services. Any sexual transaction that occurs is at the discretion of the female EE worker, and occurs elsewhere such as at a nearby hotel. There are also instances where the female EE workers could have casual sex with the men [22, 23].

This raises an important public health concern, as there is evidence to suggest that the STI prevalence rate among the female EE workers is much higher than that of the brothel-based sex workers (20.7 % versus 5.0 % respectively between 2007 and 2009) [22]. The aim of this study was to compare the prevalence and factors associated with risky sexual behaviours among the heterosexual men who patronised the EEs and brothels in Singapore.

## Methods

### Study design and population

This was a cross-sectional survey conducted between December 2011 and October 2012 among heterosexual male residents in Singapore. Two groups of heterosexual men were recruited from the licensed brothels and the EEs respectively. Study participants had to be heterosexual males between 21 and 70 years old who reported engaging in vaginal, oral or anal sex either with a female sex worker or a female EE worker in the past 12 months preceding the survey. To detect a difference of at least 15 % in the proportion of consistent condom use between the two groups, we estimated the sample size to be about 300 respondents per group to give a power of 80 % and a level of significance (alpha) of 0.05 (two sided) assuming a participation rate of 70 % based on a previous survey by the research team.

### Sampling frame and ethics approval

A 2-stage sampling strategy was employed to obtain the sample of the heterosexual men in both the groups: proportional stratified random sampling of the venues, followed by time location sampling of the men. The sampling frame of licensed brothels (*n* = 93) which came from the designated red-light districts was obtained from the Department of STI Control. For the sampling frame of EEs (*n* = 556), this was obtained from the Singapore Police Force and updated with our geographical reconnaissance work. This involved field visits by the research team to exclude venues that have closed down and to include newly opened ones. The EEs came from five different geographical areas in Singapore. From these two sampling frames, proportional stratified random sampling was performed with 61 brothels and EEs each selected for the study, taking into account non-response. Next, five heterosexual male clients from each of the 61 brothels and EEs respectively were selected using time location sampling. This meant that men were selected using systematic sampling at different times of the day during the venues’ operating hours from 12 noon to 12 midnight and over weekdays and weekends over a 4-month period to reduce selection and seasonal bias. The time location sampling was continued until the target sample size had been reached. Although there was a possibility that a man could have participated multiple times in the survey if he had visited multiple settings, this was minimised through time location sampling and training of our interviewers to identify duplicate participants by asking whether they had participated in the survey before. We did not find any duplicate participants. Interviewers approached 310 men each from the brothels and EEs as they stepped out from these venues. The response rate was 96.8 % for the brothels and 90.6 % for the EEs. Two hundred ninety seven men from the brothels and 272 men from the EEs were considered for the final analysis after excluding three and nine men respectively from each group due to incomplete data or not fitting the eligibility criteria. The study was approved by the National University of Singapore Institutional Review Board.

### Data collection

Interviewers, working in pairs for safety reasons, waited outside the randomly selected brothels or the EEs and requested interviews from men who stepped out from these places. The following measures were taken to increase response and minimise self-reporting bias. Firstly, we engaged students from local tertiary institutions to be interviewers as they would likely be perceived to be less threating than official staff. They were trained to approach the men in a polite, non-threatening and non-judgemental manner. Secondly, the interviewers explained to the men that they were conducting a lifestyle survey to help plan a programme to improve their health. They also reassured the men that no personal identifiers such as their names or contact details would be collected and that the responses would be kept strictly anonymous and confidential. Thirdly, the interviewers put the men at ease by starting with informal conversations before asking non-sensitive questions on their age and resident status to identify whether they fit the eligibility criteria. The men were then asked to read the next question (also translated into Chinese and Malay) and ticked the types of sex partners they had sex with in the past 12 months. If they ticked ‘wife’ and/or ‘girlfriends’ only, the interviewer then stopped the interview, thanked them and gave them a small gift (tissue paper packs with health messages and a canned drink). If they ticked ‘casual sex partners’ (defined as a partner who was not paid for sex and who was not a regular partner or in a longstanding sexual relationship with the participant) or ‘prostitutes’, the interviewer then asked them to complete a short questionnaire after showing them the participant information sheet and obtaining oral consent.

### Statistical analyses

All statistical analyses were performed using STATA version 11.2 (Stata Corp, College Station, TX). To describe the socio-demographic characteristics and sexual behaviour of the men who patronised EEs and brothels respectively, we compared categorical variables with the use of the χ^2^ test and continuous variables with the independent-sample t test. *P* value of ≤0.05 was taken as statistically significant. The whole dataset was then used to assess the independent factors associated with consistent condom use in the heterosexual men. These factors were socio-demographic characteristics, setting type, partner type and sexual behaviour characteristics. A mixed effects logistic model was used to account for clustering by venue. We obtained the crude OR and 95 % CI for the association of each of these factors with consistent condom use for vaginal and oral sex respectively. To further identify the independent factors, all the factors with p value ≤0.10 were entered into each of the 2 models of consistent condom use for vaginal and oral sex. We then performed multivariable analysis with a backward elimination procedure to obtain the adjusted odds ratio (aOR) and 95 % CI for consistent condom use in vaginal and oral sex respectively.

## Results

Table 1 compares the socio-demographic characteristics of men who patronised brothels and EEs. Men who patronised EEs were younger, more likely to be single and of higher socio-economic status, in that 22.4 % of them stayed in private property/condominiums/bungalows compared to 9.4 % in the brothel group. They were also more highly-educated, with 64.8 % of them having completed tertiary and university education compared to 26.9 % of men who patronised the brothels. There were also a greater proportion of professionals, businessmen and managers in the EE group (33.1 %) compared to the brothel group (16.2 %).

**Table 1:** Comparison of socio-demographic characteristics of men who patronised brothels and entertainment establishments

Characteristic	Brothels (*N* = 297)	Entertainment Establishments (*N* = 272)	p value*
	N (%)	N (%)	
Age (years)			
Mean (SD)	44.5 (12.5)	34.9 (10.5)	
Median	47.0	33.0	<0.001
Range	21-66	21-64	
Ethnicity			
Chinese	263 (88.6)	200 (73.5)	
Malay	17 (5.7)	16 (5.9)	
Indian	13 (4.4)	47 (17.3)	<0.001
Others	3 (1.0)	9 (3.3)	
Unknown	1 (0.3)	0	
Marital Status			
Single	158 (53.2)	165 (60.7)	
Married	90 (30.3)	89 (32.7)	
Divorced	43 (14.5)	16 (5.9)	0.003
Widowed	1 (0.3)	0	
Unknown	5 (1.7)	2 (0.7)	
Housing			
1–3 room public housing	151 (50.9)	82 (30.2)	
4–5 room public housing	115 (38.7)	129 (47.4)	<0.001
Private Property/ Condominium/ Bungalows	28 (9.4)	61 (22.4)	
Unknown	3 (1.0)	0	
Education			
Primary or less	113 (38.1)	26 (9.5)	
Secondary	95 (32.0)	67 (24.6)	
Tertiary^a^ (excludes university & post-graduate)	61 (20.5)	119 (43.8)	<0.001
University & post-graduate	19 (6.4)	57 (21.0)	
Unknown	9 (3.0)	3 (1.1)	
Occupation			
Professional, businessmen, managers	48 (16.2)	90 (33.1)	
Technicians, service staff, taxi drivers	77 (25.9)	98 (36.0)	<0.001
Labourers, construction workers	74 (24.9)	11 (4.0)	
Others	98 (33.0)	73 (26.9)	

*Excludes unknown values

^a^Includes institute of technical education, junior college and polytechnic level of education

On comparing their sexual behaviours in the past year (Table 2), the prevalence of casual sex was higher in the EE group (78.3 %) than in the brothel group (26.3 %) although their median number of casual partners was similar. For paid sex, the prevalence was higher in the brothel group (91.9 %) compared to the EE group (37.1 %). The median number of paid sex partners was also higher in the brothel group compared to the EE group. The EE group reported higher rates of vaginal, oral and anal intercourse with both casual and regular (wife/girlfriend) partners than the brothel group. Condom use varied by the type of partner. Consistent condom use for vaginal intercourse was highest with sex workers, decreased with casual partners and was lowest with wife/girlfriend in both groups. On comparing condom use by the type of sexual intercourse, both groups reported the lowest rates of condom use for oral sex with all partner types. Consistent condom use for oral sex with sex workers was significantly lower in the EE group (26.6 %) than in the brothel group (56.6 %). Consistent condom use for vaginal sex with casual partners was also significantly lower in the EE group (40.7 %) than in the brothel group (53.9 %). In addition, men who patronised the EEs (28.0 %) were less likely than the brothel group (44.4 %) to screen for HIV/STI in the past year although their rates of condom use were consistently lower with all types of partners and for all types of intercourse compared to the brothel group except for anal intercourse with wives/girlfriends and sex workers.

**Table 2:** Comparison of sexual behaviours of men who patronised brothels and entertainment establishments

Sexual behaviour	Brothels (*N* = 297)	Entertainment Establishments (*N* = 272)	*p* value
	N (%)	N (%)	
Ever engaged in paid sex in the past 12 months	273 (91.9)	101 (37.1)	<0.001
Ever engaged in casual sex in the past 12 months	78 (26.3)	213 (78.3)	<0.001
Numbers of partners in the past 12 months			
Sex workers			
Mean(SD)	9.1 (17.6)	3.8 (4.5)	0.003
Median (range)	3 (1–186)	2 (1–24)	<0.001
Interquartile range	2–10	1–4	
Casual partners			
Mean(SD)	3.3 (4.1)	4.1 (17.5)	0.67
Median (range)	2 (1–20)	2 (1–250)	0.81
Interquartile range	1–12	1–16	
Types of sexual intercourse in the past 12 months			
Sex workers			
Vaginal	271 (91.2)	101 (37.1)	<0.001
Oral	212 (71.4)	64 (23.5)	<0.001
Anal	72 (24.2)	20 (7.4)	<0.001
Casual partners			
Vaginal	76 (25.6)	204 (75.0)	<0.001
Oral	68 (22.9)	160 (58.8)	<0.001
Anal	21 (7.1)	54 (19.9)	<0.001
Wife/girlfriend			
Vaginal	129 (43.4)	159 (58.5)	<0.001
Oral	93 (31.3)	133 (48.9)	<0.001
Anal	33 (11.1)	46 (16.9)	0.05
Consistent condom use in the past 12 months			
Sex workers			
Vaginal	195 (72.0)	68 (67.3)	0.38
Oral	120 (56.6)	17 (26.6)	<0.001
Anal	43 (59.7)	13 (65.0)	0.67
Casual partners			
Vaginal	41 (53.9)	83 (40.7)	0.04
Oral	24 (35.3)	49 (30.6)	0.50
Anal	14 (66.7)	33 (61.1)	0.65
Wife/girlfriend			
Vaginal	33 (25.6)	34 (21.4)	0.40
Oral	23 (24.7)	26 (19.5)	0.35
Anal	14 (42.4)	27 (58.7)	0.15
Screened for HIV/STI in the past 12 months	132 (44.4)	75 (28.0)	<0.001
Self-reported STI symptoms	15 (5.0)	8 (3.0)	0.20

Table 3 shows the association of the various factors with consistent condom use in vaginal sex. On multivariable analysis, this decreased at the EE setting (aOR 0.64; 95 % CI: 0.42 – 0.97) and with alcohol use before sex (aOR 0.67; 95 % CI: 0.46 – 0.98) and increased with perceived high risk of getting HIV/STIs from partner (aOR 2.08; 95 % CI: 1.30 – 3.32) and partner’s request for condom use (aOR 5.48; 95 % CI: 1.20 –25.11). Table 4 shows the association of the various factors with consistent condom use in oral sex. On multivariable analysis, this decreased at the EE setting (aOR 0.64; 95 % CI: 0.39 – 0.98) and with alcohol use before sex (aOR 0.50; 95 % CI: 0.31 – 0.81) and increased with partner’s request for condom use (aOR 5.19; 95 % CI: 1.38 – 19.57).

**Table 3:** Crude and adjusted analysis of consistent condom use for vaginal sex with the various factors

Factor	Crude	Adjusted^a^
Venue type		
Brothel	Referent	
Entertainment Establishment	0.45 (0.32 – 0.64)	0.64 (0.42 – 0.97)
Type of partner		
Sex worker	Referent	
Casual partner	0.59 (0.28 – 1.23)	–
Age group (years)		
20–39	Referent	
40–70	1.20 (0.81 – 1.77)	–
Ethnicity		
Non-Chinese	Referent	
Chinese	1.65 (0.69 – 3.95)	–
Marital status		
Ever married	Referent	
Single	0.70 (0.40 – 1.22)	–
Housing		
1-3 room public housing	Referent	
4-5 room public housing	1.20 (0.56 – 2.57)	–
Private Property/ Condominium/ Bungalows	2.67 (1.07 – 6.68)	–
University education		
No	Referent	
Yes	1.89 (0.79 – 4.50)	–
Alcohol use before sex		
No	Referent	
Yes	0.62 (0.43 – 0.89)	0.67 (0.46 – 0.98)
Perceived high risk of getting HIV/STIs		
No	Referent	
Yes	1.66 (1.10 – 2.50)	2.08 (1.30 – 3.32)
Partner asked to use condoms all the time		
No	Referent	
Yes	5.14 (1.13 – 23.39)	5.48 (1.20 – 25.11)

^a^In the multivariable analysis model, all crude variables with a p value of ≤0.1 are entered into the model. Only variables which are statistically significant on the final multivariable analysis are shown in the “Adjusted” column of the table

**Table 4:** Crude and adjusted analysis of consistent condom use for oral sex with the various factors

Factor	Crude	Adjusted^a^
Venue type		
Brothel	Referent	
Entertainment Establishment	0.40 (0.27 – 0.60)	0.64 (0.39 – 0.98)
Type of partner		
Sex worker	Referent	
Casual partner	0.78 (0.29 – 2.05)	–
Age group (years)		
20–39	Referent	
40–70	1.68 (1.09 – 2.59)	–
Ethnicity		
Non-Chinese	Referent	
Chinese	1.14 (0.40 – 3.26)	–
Marital status		
Ever married	Referent	
Single	0.66 (0.44 – 0.99)	–
Housing		
1-3 room public housing	Referent	
4-5 room public housing	1.26 (0.49 – 3.28)	–
Private Property/ Condominium/ Bungalows	2.44 (0.77 – 7.73)	–
University education		
No	Referent	
Yes	1.61 (0.53 – 4.85)	–
Alcohol use before sex		
No	Referent	
Yes	0.54 (0.36 – 0.81)	0.50 (0.31 – 0.81)
Perceived high risk of getting HIV/STIs		
No	Referent	
Yes	1.50 (0.98 – 2.34)	–
Partner asked to use condoms all the time		
No	Referent	
Yes	5.64 (1.50 – 21.27)	5.19 (1.38 – 19.57)

^a^In the multivariable analysis model, all crude variables with a p value of ≤0.1 are entered into the model. Only variables which are statistically significant on the final multivariable analysis are shown in the “Adjusted” column of the table

**Fig. 1:**
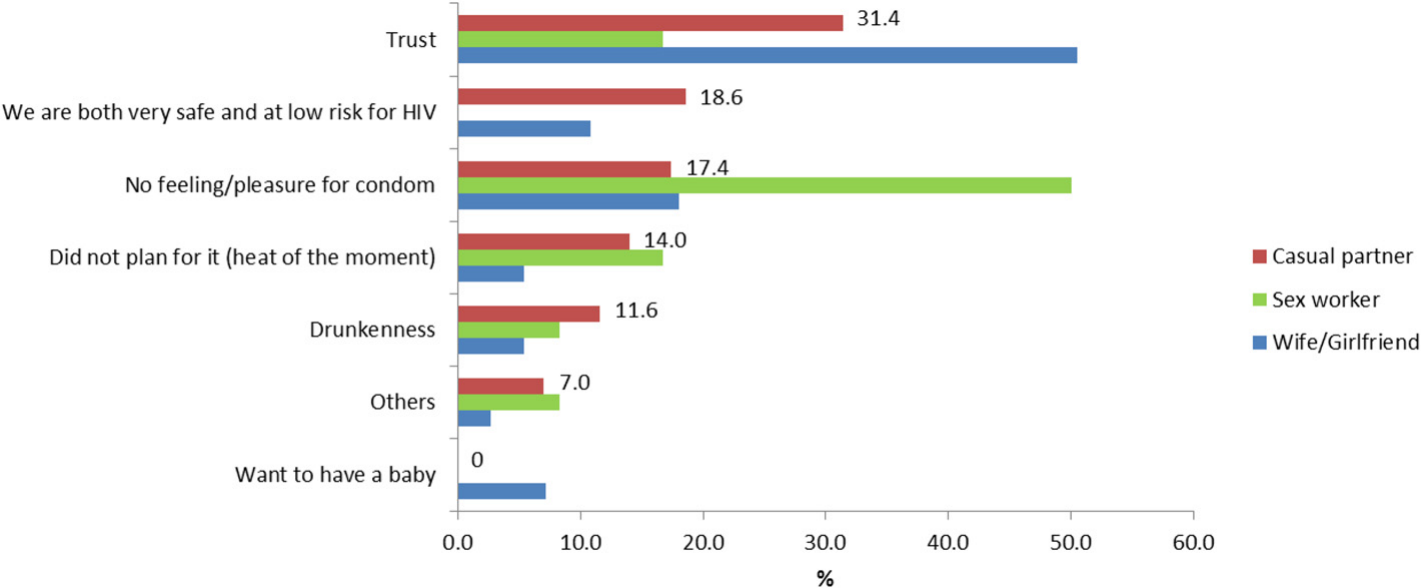
Reasons for unprotected last sex with different partner types for men who patronised entertainment establishments (*n*=272)

**Fig. 2:**
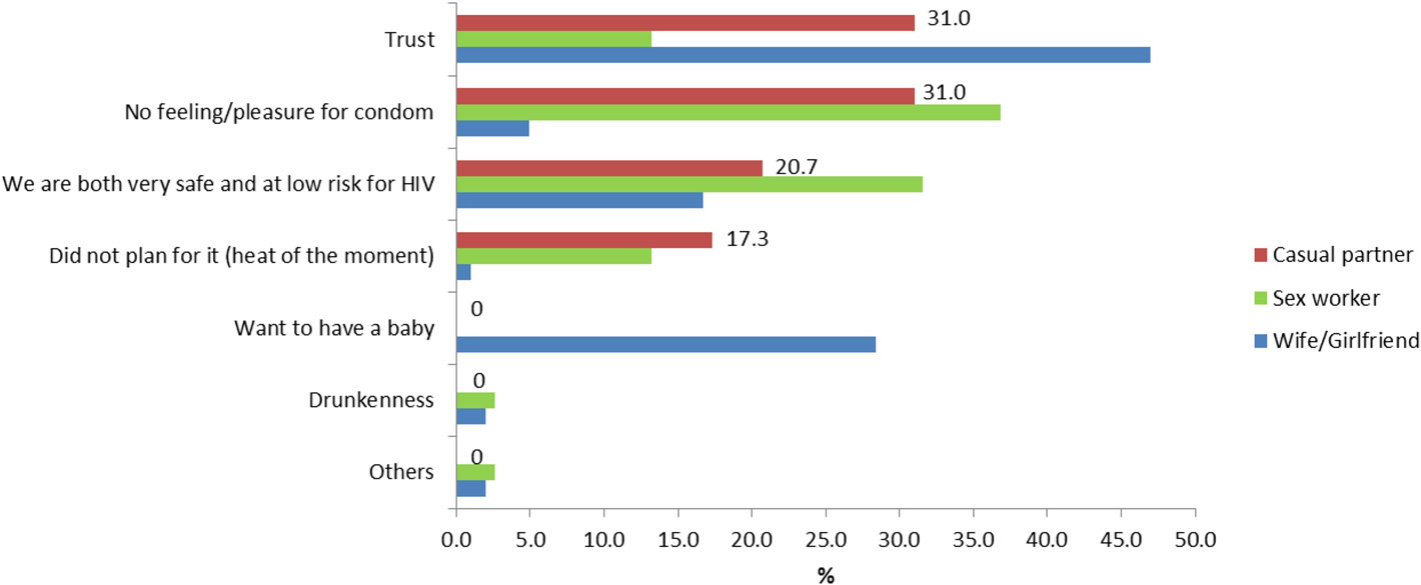
Reasons for unprotected last sex with different partner types for men who patronised brothels (*n*=297)

The reasons for unprotected last sex with different partner types are shown for the EE group (Fig. 1) and the brothel group (Fig. 2). The most common reason for not using condoms with wife/girlfriend was because of trust in both the brothel (47.0 %) and EE (50.5 %) groups. With casual partners, the most common reason for not using condoms was trust and the lack of pleasure (both 31.0 %) for the brothel group and trust for the EE group (31.4 %). Drunkenness was another reason for not using condoms with casual partners in the EE group (11.6 %) although this was not cited as a reason for the brothel group. With sex workers, the most common reason for not using condoms was the lack of pleasure in both the brothel (36.8 %) and EE (50.0 %) groups. Similarly, drunkenness was another reason for not using condoms with sex workers in the EE group (8.3 %) although this was not cited as a reason for the brothel group.

## Discussion

The EE group reported higher rates of vaginal, oral and anal intercourse with casual partners, but lower rates of consistent condom use for all three types of intercourse compared to the brothel group in the past 12 months. Consistent condom use for vaginal sex was highest with sex workers, decreased with casual partners and was lowest with wife/girlfriend in both groups. Despite riskier sexual practices with casual partners, HIV/STI screening in the past 12 months for the EE group was significantly lower compared to that of the brothel group. In multivariable analysis, consistent condom use for both vaginal and oral sex decreased in the EE setting and increased with partner’s request for condom use. Drunkenness was one of the reasons for not using condoms with both casual partners and sex workers in the EE group, although this was not cited as a reason for the brothel group.

The socio-demographic characteristics of men who patronised EEs in our study were similar to that of other studies [9–11]. In general, they were more likely to be younger, single, of higher educational level and higher socio-economic status compared to men who did not [9–11]. However there were also important differences in the sexual behaviour of men who patronised EEs in our study compared to the others. For example, among the men who patronised the EEs in Tijuana, Mexico in 2008 and had paid vaginal or anal sex with the female EE workers, 50.3 % of them did not use condoms [10]. This was 32.7 % and 35.0 % for paid vaginal and anal sex respectively in our study. Men who patronised EEs in our study reported a seemingly much higher prevalence of casual sex at 78.3 % for the past one year compared to 35.1 % in Tijuana, Mexico [10] and 24.8 % in Liuzhou, China [11]. In addition, alcohol use was previously reported to be associated with paid sex for men who patronised EEs in Tijuana, Mexico (OR 3.12; 95 % CI: 1.54 – 6.30) [10] and our study filled the gap in knowledge by showing that alcohol use also decreased consistent condom use for oral sex (aOR 0.57; 95 % CI: 0.38 –0.87).

Despite the higher education level and greater proportion of professionals in the men who patronised the EEs compared to the brothels, this did not increase their likelihood of condom usage. There are a few possible reasons for this. Firstly, while traditional brothels often have in place HIV/STI prevention programmes in the form of easy access to condoms, screening and treatment services [17–21], these are minimal in EEs. The years of educational efforts and various programmes targeting sex workers in the brothels, in addition to the medical surveillance programme where all sex workers in licensed brothels are required to go for mandatory STI screening, and the 100 % condom usage policy in all licensed brothels in Singapore, have promoted the widespread use of condoms among these sex workers [17, 18]. Studies have found that consistent condom use was generally high for ‘regulated’ commercial sex, reaching up to more than 90 % among the brothel-based sex workers [18]. Men in the brothel group could have ‘learnt’ the behaviour of consistent condom use during sex with sex workers, and this similar behaviour could then have been adopted during vaginal sex with the casual partners. Secondly, in contrast to licensed brothels, commercial sex transaction in EEs is illegal in Singapore and often occurs under the table; therefore, many EE workers hesitate to carry condoms with them because of the fear that they could be used as circumstantial evidence for prostitution by the police. Thirdly, alcoholic drinks are more commonly consumed in the EEs than in the brothels in Singapore [24]. Our study showed that consistent condom use for both vaginal and oral sex decreased with alcohol use before sex in the multivariable analysis and that drunkenness was one of the reasons for unprotected sex with both casual partners and sex workers in the EE group but not the brothel group. Alcohol use in sexually stimulating environments, such as the EEs, is associated with an increased likelihood of unprotected sexual intercourse [25]. Heavy alcohol use in men before sex has been previously reported to be significantly associated with inconsistent condom use over the last year (aOR 2.40; 95 % CI: 1.21 – 4.77) [26]. Therefore, increased alcohol consumption before sex may have contributed to the lower rates of consistent condom use observed in the EE group. Fourthly, different perceptions and expectations by the heterosexual men towards the female workers in the EEs and brothels might be another possible reason for the differences in condom use. Compared to men who visit brothels for paid sex, not all men who patronise EEs might have the intention of engaging in sex with the female EE workers. They might be visiting these venues for a drink, to sing and dance or to socialise with their friends. Therefore the sexual encounter in the EE setting might not always be a planned event and hence the men would not necessarily come prepared with a condom, particularly in instances of casual sex with female EE workers.

Men who patronised EEs reported the lowest level of consistent condom use with wives/girlfriends among their sexual partners for all types of intercourse and the most common reason given was trust. This low level of condom usage, in addition to the low level of HIV/STI screening in men who patronised EEs, raises the public health concern that these men could potentially act as a bridging population for HIV/STI transmission through unprotected sex with the sex workers, female EE workers or casual partners to their spouses or regular partners. Clearly, it is important to reach out to these men through the EE setting where both casual and paid sex with the female EE workers is common. Intervention programmes should address the reasons for unprotected sex with casual and paid partners, emphasise how over-consumption of alcohol could lead to risky sexual behaviour and inform on available screening facilities.

Other than men, it is also important to target the female EE workers. Our results have shown that most of the reasons for condom use in men who patronised the EEs were extrinsic in nature, and that an important independent factor of consistent condom use for both vaginal and oral sex was partner’s request for condom use. This is also similar to a previous study among Singaporean men patronising female sex workers abroad and locally, where condom use was found to be determined more by extrinsic factors such as the sex workers’ actions rather than the clients’ [27].

Programmatic recommendations to the EE settings are not so clear-cut, but there is a growing consensus that the interventions should be structural and targeting all key stakeholders, including the owners and management, for successful HIV/STI prevention [28–33]. In a study in the southern Philippines [28], female EE workers who received peer and establishment manager interventions, and establishment policies favouring condom use, reported more positive condom attitudes and fewer STIs than the peer-only intervention and the control group. Besides health education on STI and HIV knowledge, it is also important to emphasise condom use for both casual and paid partners in a non-discriminating manner to reduce stigmatisation, as well as provide communication training to enhance the female EE workers’ skills in condom negotiation with their male partners [34]. Other than condom promotion, STI screening and case finding are also important for decreasing HIV/STI transmission [35–37]. In the Philippines, a network of social hygiene clinics provides STI screening and treatment for registered female EE workers in over 140 cities for over 1000 women weekly, where HIV prevalence has remained relatively low among the female sex workers compared to the other countries in the region [38]. However, other studies have suggested that the low HIV rates in the female workers might be due to lower exposure to risk (e.g., lower numbers of clients per week/month and shorter duration of sex work) rather than to preventive interventions [31]. It was also reported that the recommended programmatic actions tried in different settings did not necessarily produce good results all the time [32]. This was particularly so when stakeholder engagement was poor, for example in the case when owners and pimps obstructed such preventive interventions to the sex workers [33].

Implementation of preventive interventions in Singapore will be challenging given that commercial sex is currently illegal in the EEs. In light of the political and legal sensitivities, preventive interventions could focus on promoting general sexual well-being as part of workplace health promotion in the EEs. A multi-sectorial approach with the engagement of the EE management and relevant government and non-governmental agencies is needed to promote safer sex behaviours and increase access to HIV/STI screening services.

### Strengths and limitations

This study has some limitations. The two groups may not be mutually exclusive because men who patronised the EEs may have also visited brothels and vice versa. This spill-over rate is estimated at about 10 % from an earlier study that was conducted [22], and it is unlikely to have any significant impact on the findings. In addition, restricting our analysis to men who only patronised either the EEs or the brothels alone showed similar findings. Another limitation might be social desirability bias on self-reporting of condom use among participants. However, this was expected to be minimal in view of the low rates of condom use across both groups. In addition, we tried to facilitate recall by approaching men who had just stepped out of the brothels and EEs.

To date, this is to our knowledge the first study in an Asian urban setting to compare the risky sexual behaviours of heterosexual men who patronise EEs and brothels. The study fills the current gaps in knowledge pertaining to casual sex behaviour and oral intercourse practices between the two groups of men. The samples of men for both the groups were obtained from a sampling frame of brothels and EEs. Careful sampling procedures were applied to ensure a representative random sample so that the findings could be generalised to the population of heterosexual male residents who engage in paid and casual sex in brothels and EEs. The high response rate and use of time location sampling also supports the generalisability of the study findings to all the licensed brothels and EEs in Singapore.

## Conclusions

Men who patronised EEs practised riskier sexual behaviours compared to men who visited brothels. The low condom usage with both paid and casual partners warrants prompt interventions to prevent HIV/STI transmission. Priority should be given for intervention programmes to target men who patronise EEs, which could involve the female EE workers, the EE owners as well as the managers for effective HIV/STI prevention.

## Abbreviations

aOR: adjusted odds ratio
CI: Confidence interval
EEs: Entertainment establishments
HIV: Human immunodeficiency virus
OR: Odds ratio
STIs: Sexually transmitted infections
